# Spillover of Canine Parvovirus Type 2 to Pigs, South Dakota, USA, 2020

**DOI:** 10.3201/eid2802.211681

**Published:** 2022-02

**Authors:** Gun Temeeyasen, Tamer A. Sharafeldin, Chun-Ming Lin, Ben M. Hause

**Affiliations:** South Dakota State University, Brookings, South Dakota, USA

**Keywords:** parvovirus, canine parvovirus type 2, CPV-2, spillover, pig, viruses, South Dakota

## Abstract

In 1978, canine parvovirus type 2 originated from spillover of a feline panleukopenia–like virus, causing a worldwide pandemic of enteritis and myocarditis among canids. In 2020, the virus was identified in pigs in South Dakota, USA, by PCR, sequencing, in situ hybridization, and serology. Genetic analysis suggests spillover from wildlife.

Canine parvovirus type 2 (CPV-2) is a variant of the species *Carnivore protoparvovirus 1*, which can cause severe disease in carnivores of many species (*1*–*3*). Besides CPV-2, which causes enteritis in dogs of all ages and myocarditis in puppies, the virus species includes feline panleukopenia virus, which causes severe enteritis and leukopenia in cats of all ages (*4*). In 1978, CPV-2 emerged and caused a worldwide pandemic after spillover from a feline panleukopenia virus–like virus in wildlife. Subsequent adaptation to canine hosts led to genetic and antigenic diversification into subtypes 2a, 2b, and 2c (*5*). Continued CPV host switching has been documented; spillover to wildlife (including skunks, raccoons, coyotes) has resulted in clinical disease and asymptomatic infection (*2*).

In October 2020, a dead pig was submitted to South Dakota State University (Brookings, SD, USA) for diagnostic testing. Histopathologic examination revealed mild to moderate enteritis, hepatitis, and visceral edema. Hemolytic *Escherichia coli* was isolated. No significant lung lesions were noted. Approximately 8 months later, we performed viral metagenomic sequencing on archived lung tissue for an unrelated research project and unexpectedly identified CPV-2. Using a S′ nuclease PCR (Integrated DNA Technologies, https://www.idtdna.com), we confirmed that the sample was CPV-2 positive; cycle threshold (C_t_) was 24.4. Sanger sequencing of overlapping amplicons confirmed the CPV-2 genome sequence determined by metagenomic sequencing. We submitted the strain SDS21601 sequence to GenBank (accession no. MZ666397).

We used a S′ nuclease PCR to test 90 archived porcine lung samples submitted for respiratory disease diagnostic testing for CPV-2. Of the 90 samples, 9 (10%) were positive for CPV-2, including those with strain SDS21601, and C_t_ values were 22.4–36.3. The samples were collected September–November 2020 from swine farms within 150 miles of Brookings. We sequenced the genome from a second strongly positive sample (C_t_ 22.4) and submitted strain SDS21608 to GenBank (accession no. MZ666398). An amplicon from 4 of the remaining 7 samples positive by PCR was generated by PCR and confirmed as CPV-2 by Sanger sequencing. The 3 samples that failed to yield a CPV-2–specific amplicon had C_t_ values >32. Sequence comparison showed 99.9% nt identity between SDS21601 and SDS21608. blastp (https://blast.ncbi.nlm.nih.gov) analysis of SDS21601 virus capsid protein (VP) 2 found 100% identity to CPV-2 from a coyote sampled in Montana in 2012. Analysis of the VP2 amino acid sequences identified an F212I mutation previously identified only from US wildlife, mainly coyotes.

We performed in situ hybridization on archived formalin-fixed paraffin-embedded tissues from SDS21608 by using a commercially available CPV-2 probe. CPV-2 nucleic acids were hybridized sporadically as intracytoplasmic punctate signals in few monocyte–macrophage lineage cells in the medullary and subcapsular sinuses of a bronchial lymph node (Figure). However, the primary anatomic site of CPV-2 infection and replication was not determined. In other examined tissues, we observed neither typical virus-associated microscopic lesions as seen in carnivores nor obvious CPV-2 hybridization signals.

**Figure:**
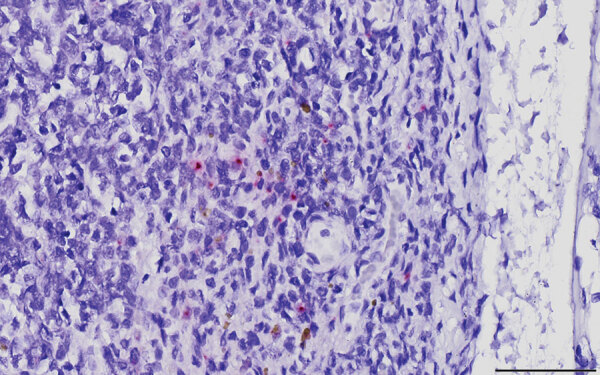
Canine parvovirus type 2 (CPV-2) nucleic acid in a bronchial lymph node obtained from a commercial pig, South Dakota, USA, 2020. Virus was detected by in situ hybridization with a commercially available CPV-2 probe (Advanced Cell Diagnostics, https://acdbio.com). Signals of CPV-2 nucleic acid were hybridized as intracytoplasmic red pinpoints in a few cells morphologically resembling monocyte–macrophage lineage cells in the medullary sinus. Golden-brown pigments are suggestive of hemosiderin accumulated in the cytoplasm of macrophages. The slide was counter-stained with hematoxylin. Scale bar indicates 50 μm.

To further investigate the extent of CPV-2 circulation among swine, 8 months after collection of the CPV-2–positive lung tissue, we collected 20 serum samples from multiparous sows on the farm where strain SDS21601 originated. Of the 20 samples, 13 (65%) were positive for CPV-2–specific antibodies by hemagglutination inhibition (HI) assay; titers were 10–40 (Table). Nearly all serum samples (19 of 20) had antibody titers to porcine parvovirus 1 (PPV-1), ranging from 16 to 4,096. This result was expected given that pigs on the farm received commercial PPV-1 vaccine before farrowing. There was no correlation between CPV-2 and PPV-1 HI titers, indicating a lack of cross-reactivity between CPV-2 and PPV-1 antibodies in the HI assay. 

**Table:** Antibody titers for CPV-2 and PPV-1 in serum collected from multiparous sows at origin farm of CPV-2 strain SDS21601 ≈8 months after collection of CPV-2–positive lungs, South Dakota, USA, 2020

Sow no.	CPV-2 titer	PPV-1 titer
3818	20	16
8985	10	256
3407	20	1024
4344	0	256
4345	10	512
3406	0	2048
3410	0	4096
37681	10	4096
38679	20	4096
39692	0	1024
4347	20	64
37683	20	2048
37673	40	2048
8980	0	256
445	10	512
8952	10	512
8953	0	2048
8981	20	1024
3817	20	0
10040	0	128

*Determined by hemagglutination inhibition**.** CPV-2, canine parvovirus type 2; HI, hemagglutination inhibition; PPV-1, porcine parvovirus type 1.

To further investigate seroprevalence of CPV-2 in South Dakota, we randomly selected 25 sow serum samples from unrelated submissions collected at 5 farms (5 samples/farm) and analyzed them by HI for CPV-2. Of the 25 samples, 23 (92%) were positive for CPV-2; titers were 10–80. Together with the 10% positivity detected by quantitative PCR, these results suggest widespread CPV-2 infection of swine in South Dakota.

Members of *Carnivore protoparvovirus 1* display >98% identity. Amino acid residue 300 of VP2 has been shown to be a critical determinant for the cross-species transfer of CPV-2 between carnivores of different species (*6*). Glycine 300 and tyrosine 305, observed in the VP2 of both swine CPV-2 strains (SDS21601 and SDS21608), are diagnostic of CPV-2 isolates from canids (*7*). The F212I mutation present in both swine CPV-2 strains, which was previously found only in wildlife, suggests a sylvatic origin. Of the species in which F212I has been identified, only coyotes are common in the agricultural areas of the upper US Midwest and are peridomestic. We hypothesize that the source of swine CPV-2 infection is CPV-2–positive coyote feces.

Our results demonstrate spillover of CPV-2 to swine. CPV-2 has been associated with severe enteritis in insectivorous Taiwanese pangolin (*Manis pentadactyla pentadactyla*), further demonstrating the propensity of CPV-2 to overcome host barriers (*8*). The ability of CPV-2 to cause disease in swine remains unknown; further surveillance is warranted because this spillover may threaten the health of swine herds.
